# Supplementary material for: Microbes and diet reshape the intestine via distinct cellular dynamics
Source: bioRxiv. 2025 Sep 17:2025.09.17.676798. Preprint. [Version 1] doi: 10.1101/2025.09.17.676798 (PMC12458162; doi:10.1101/2025.09.17.676798)
Supplement: 1 [file NIHPP2025.09.17.676798v1-supplement-1.pdf]

bioRxiv preprint doi: https://doi.org/10.1101/2025.09.17.676798; this version posted September 17, 2025. The copyright holder for this preprint (which was not certified by peer review) is the author/funder, who has granted bioRxiv a license to display the preprint in perpetuity. It is made available under aCC-BY 4.0 International license.

Supplementary materials and methods

Fly Stocks

Drosophila melanogaster stocks were maintained at room temperature (~23°C) on yeast-cornmeal medium or at 18°C under a 12:12 hr light/dark cycle. Canton-S (Cs) (BDSC: 64349), was used as the wild-type control in all experiments not involving transgenic constructs. Gal4 drivers used included: '*w-; Esg-Gal4; UAS-GFP, tub-Gal80*^{TS}' (*Esg*^{TS}, progenitor-specific) [10] and '*5966-GS*' (EC-EB-specific, RU486-dependent gene-switch) [45]. UAS lines used were UAS-Histone2B-RFP [46], *UAS-rpr*^{OE} (BDSC: 5823), and the *w*^{1118} background strain.

Experimental Design

All experiments were performed on mated female flies. Crosses and fly handling followed our previously described protocol [3], with minor adaptations. Briefly, conditional gene expression was induced using the TARGET system [47] or the GeneSwitch system (online supplemental figure 1A). For TARGET, flies developed at 18°C on a pre-experiment diet (yeast-cornmeal medium). F1 progenies were collected within 3 hours of eclosion and transferred to experimental diets. Transgenes were induced either by shifting to 29°C (TARGET) or by administering RU486 (100 μL of a 5 mg/mL solution in 80% ethanol added on top of food, with ethanol left to evaporate before adding flies) for GeneSwitch [48]. Genotype and treatment matched controls were used in all cases.

Food Production

Standard, HS, and HY diets were prepared as previously described [3]. Briefly, agar was dissolved in boiling water, followed by addition of dry ingredients while constantly stirring. Acid mix was added at ~60°C, and food was aliquoted into vials at ~40°C.

Bacterial cultures and oral infection

Bacterial cultures and oral infection were performed as previously described [18]. *Erwinia carotovora ssp. carotovora 15* (*Ecc15*)[49], its evf-deficient mutant (*Ecc15*$^{evf-}$) [43] and *Escherichia coli* (*E.coli*) were maintained on standard LB agar. To obtain bacteria for infections, single colonies were inoculated into LB broth and grown at

bioRxiv preprint doi: https://doi.org/10.1101/2025.09.17.676798; this version posted September 17, 2025. The copyright holder for this preprint (which was not certified by peer review) is the author/funder, who has granted bioRxiv a license to display the preprint in perpetuity. It is made available under aCC-BY 4.0 International license.

29 °C, shaking for 16 hrs. Cultures were then pelleted (3,000 g, 5 min), and resuspended in PBS to an optical density at 600 nm (OD_{600}) of 200.

Adult flies were starved in empty vials for 2 h at 29 °C, then transferred to vials whose standard food surface was covered with a Whatman filter paper disk saturated with 150 µL of 2.5% sucrose and bacterial suspension (final OD_{600} = 100). Flies remained at 29 °C until the designated dissection time point or survival assay endpoint.

Immunochemistry

As previously performed [3], midguts were fixed in 4% paraformaldehyde, washed in PBS with 0.1% Triton X-100, and blocked in 1% BSA and 1% normal donkey serum. Immunostaining was performed using mouse anti-Dlg1 (1:100, DHSB 4F3, AB_528203) as primary antibody and Alexa Fluor-conjugated secondary antibodies (Thermo Fisher). DNA was stained with DAPI (1:50,000) and mounted in Citifluor AF1. Imaging was performed on a Zeiss LSM 700 confocal microscope.

Posterior Midgut Area Measurements

Midgut area was quantified by capturing tiled 10× fluorescence images of whole midguts, assembled in Zen software (Zeiss). A custom FIJI [50] macro was developed and used to threshold midguts in a broad user-selected area containing the posterior region of the midgut. Binary masks were generated, holes filled, and area was quantified via particle analysis.

Cell Size Measurement

EC area was manually measured in FIJI from anti-Dlg1, stained midguts imaged as Z-stacks with a 20× objective with Zen software (Zeiss). The largest visible plane of each cell was manually outlined in FIJI using the polygon tool. Approximately 30 adjacent cells per midgut were measured, as previously performed [3].

Cell loss assay

For a visual scheme of this experiment, please refer to online supplemental figure 1A. *5966GS>Histone2B-RFP* flies developed on standard diet and were shifted at eclosion on either HS or HY diets for 5 days. Flies were then fed RU486 for 3 days, followed by 2 days without RU486. Flies on both diets were dissected before infection procedures

bioRxiv preprint doi: https://doi.org/10.1101/2025.09.17.676798; this version posted September 17, 2025. The copyright holder for this preprint (which was not certified by peer review) is the author/funder, who has granted bioRxiv a license to display the preprint in perpetuity. It is made available under aCC-BY 4.0 International license.

(Time 0), starved for 2 hours, and kept on bacteria for eight hours. After infection, flies were shifted to either HS, HY or Agar (Ag, starvation) diets and dissected at 12, 24, 48, 96 and 240 hours post initial point of infection. UC flies were not subject to any treatment, and in case of change of diet, shifted from one diet to another directly.

Confocal Z-stacks (20×) of half midgut hemispheres were acquired and RFP+ and RFP− cell numbers were measured, together with the area of the midgut in the image, to calculate density of cells per midgut. This was multiplied for the total measure of midgut area. Cell density × area × 2 (to account for half midgut) gave total cells per region. Turnover was calculated as new cells divided by lost cells. Lost cells included both RFP+ and unmarked cells, accounting for incomplete labelling on day 0. All the calculations are as previously performed [3].

Survival

For a scheme of this experiment, please refer to figure 6A. Canton-S flies developed on standard diet and were shifted at 29C from eclosion on HS diet for 5 days. Flies were then infected daily with either *Ecc15*, *Ecc15*$^{evf-}$, or *E.Coli*. Flies were also kept in unchallenged conditions or infected once with *Ecc15*. Flies were infected for 8 hours and then shifted back to HS or to Ag diet for 16 hours before the next infection. Survival was checked daily. Only female flies were scored, but flies were cohoused with male flies to ensure mated status.

Statistical analysis

Statistical analysis was performed as previously reported [3] using generalized linear mixed models using fitme from spaMM [51]. Briefly, repeats were included in the models as random effects. Models were tested for normal distribution with Shapiro–Wilk normality tests and homoscedasticity of the residuals with Brush–Pagan tests. Non normal or heteroskedastic samples were transformed to improve fit using either log, Box Cox or squared transformation (depending on what yielded samples both normal and homoscedastic). In case no transformations were able to eliminate heteroscedasticity (only for comparisons in figure 6), resid.model was added to the model to account for heteroscedasticity. Akaike information criterion was used to select the most performing model. Models were compared with ANOVA to infer significancy. Interaction between samples was also calculated with this method. To characterize

bioRxiv preprint doi: https://doi.org/10.1101/2025.09.17.676798; this version posted September 17, 2025. The copyright holder for this preprint (which was not certified by peer review) is the author/funder, who has granted bioRxiv a license to display the preprint in perpetuity. It is made available under aCC-BY 4.0 International license.

differences multiple conditions, general linear hypotheses tests were applied, using a Tukey post hoc pairwise comparisons. Survival statistics were calculated via Cox proportional hazards mixed effects model using the coxme package in R. After performing ANOVA on the model including treatment vs the model not including treatment, Tukey post hoc test was run to identify significantly different treatment groups.

bioRxiv preprint doi: https://doi.org/10.1101/2025.09.17.676798; this version posted September 17, 2025. The copyright holder for this preprint (which was not certified by peer review) is the author/funder, who has granted bioRxiv a license to display the preprint in perpetuity. It is made available under aCC-BY 4.0 International license.

Supplemental figure legends

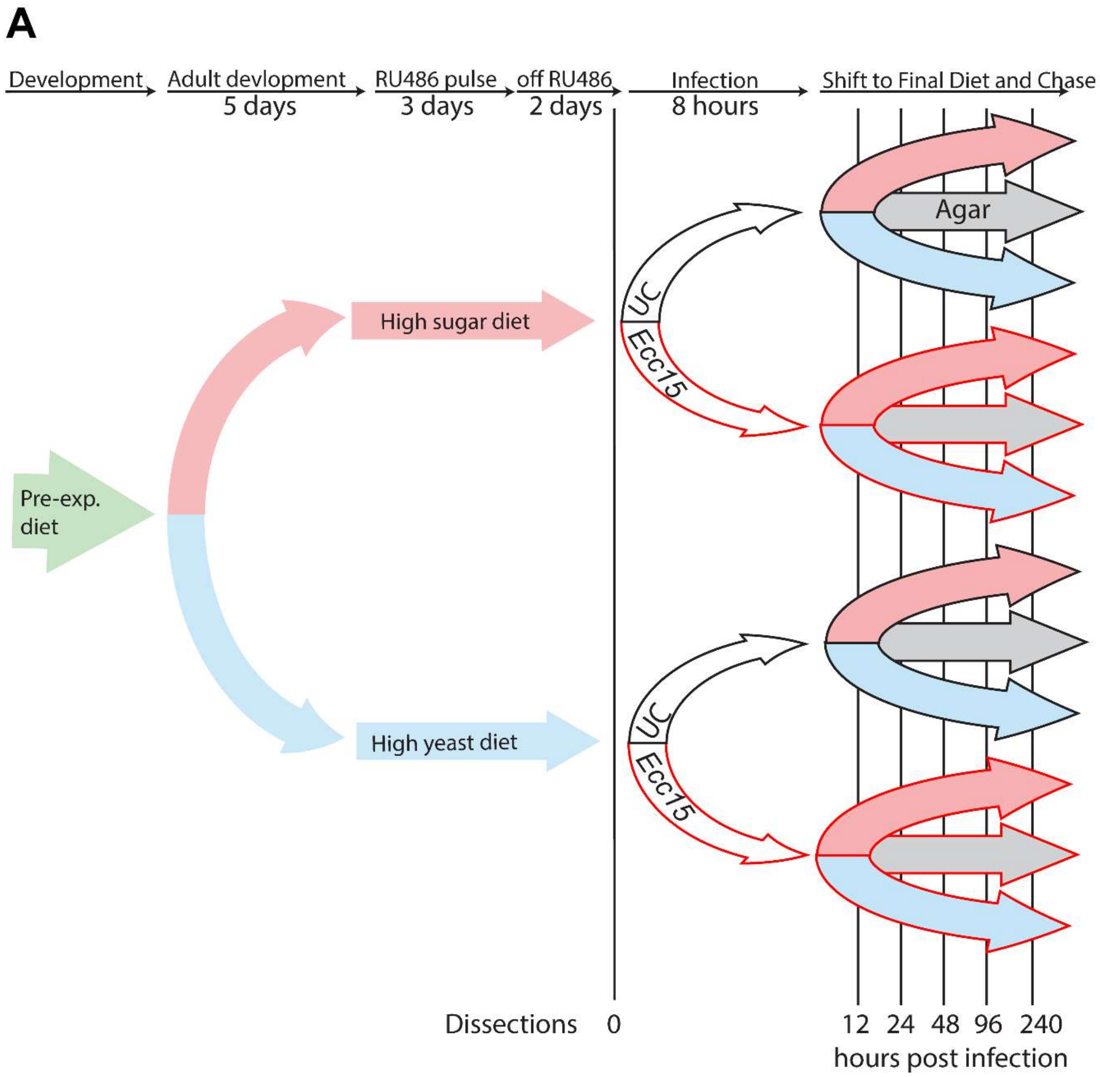

Online supplemental figure 1

(A) Scheme depicting experimental strategy. Flies are kept during development on a pre-experimental diet at 25 °C. Upon eclosion, flies are transferred to either HS or HY diet at 29 °C for 5 days to allow for development. Flies are then transferred to the same diet but containing RU486 for 3 days, in order to activate the genetic system and mark ECs present at this point in time. Flies are then transferred to the same diet without RU486 for 2 days, to allow the drug to leave the flies. Flies are then either kept infected with *Ecc15* for 8 hours or kept unchallenged (UC) and then shifted to 1) the same diet (e.g. HS to HS), 2) the other diet (e.g. HS to HY) or 3) starved (e.g. HS to Ag). Flies were either dissected pre infection, or at 12,24,48,96 and 240 hours post infection.

bioRxiv preprint doi: https://doi.org/10.1101/2025.09.17.676798; this version posted September 17, 2025. The copyright holder for this preprint (which was not certified by peer review) is the author/funder, who has granted bioRxiv a license to display the preprint in perpetuity. It is made available under aCC-BY 4.0 International license.

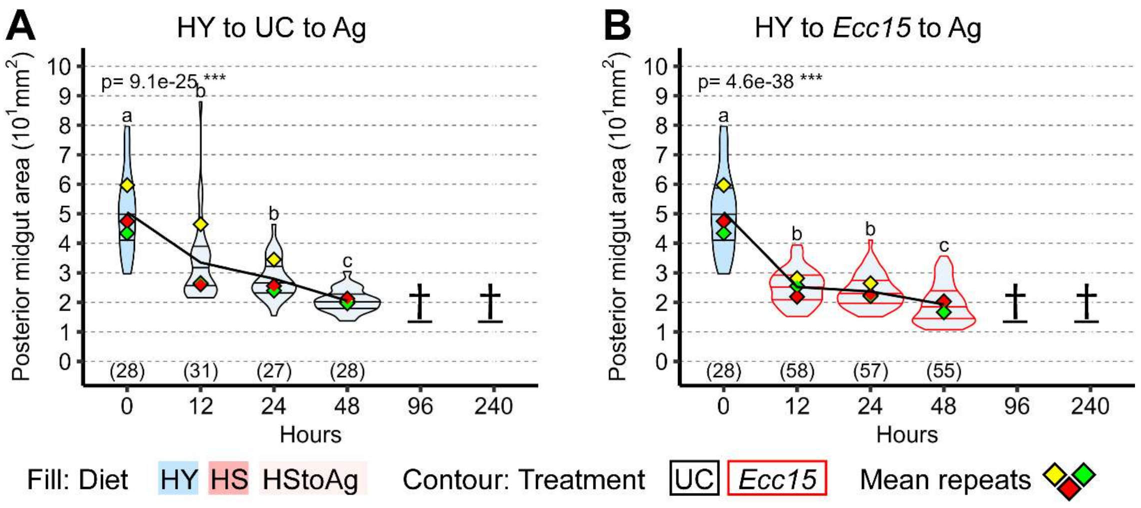

Online supplemental figure 2

(A-B) Midguts shifted from high yeast (HY) diet to agar starvation diet (Ag) in Unchallenged Conditions (UC) progressively decreased in size (A). Upon infection, midguts shrink and then maintain this small size (B). For the violin/dot plots shown in this figure, white dots represent single posterior midgut area measurements. For the violin/dot plots shown in this figure, colored lozenges represent means of replicate experiments. Black line connects total means of each sample to show timeline of changes. Violin plot fills are color-coded according to diets (HS = red, HY = light blue, HY to HS = pink, HS to HY = purple) and their contour indicates treatment (UC = black, *Ecc15* = red). Numbers in parentheses at the bottom of charts indicate sample sizes. p indicates the result of ANOVA for samples in a single chart, and groups are obtained with Tukey Post Hoc test (letters above violins).

bioRxiv preprint doi: https://doi.org/10.1101/2025.09.17.676798; this version posted September 17, 2025. The copyright holder for this preprint (which was not certified by peer review) is the author/funder, who has granted bioRxiv a license to display the preprint in perpetuity. It is made available under aCC-BY 4.0 International license.

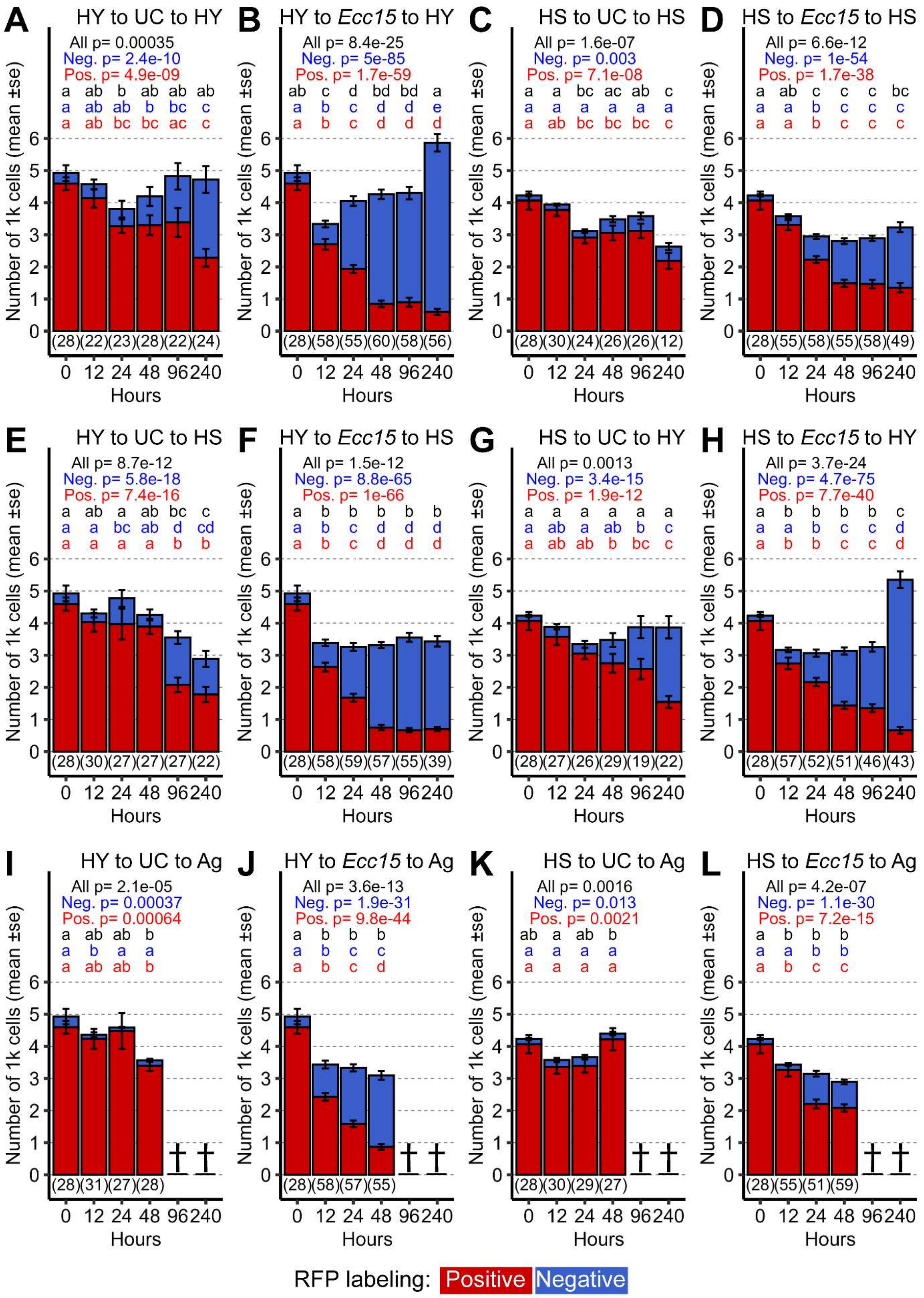

bioRxiv preprint doi: https://doi.org/10.1101/2025.09.17.676798; this version posted September 17, 2025. The copyright holder for this preprint (which was not certified by peer review) is the author/funder, who has granted bioRxiv a license to display the preprint in perpetuity. It is made available under aCC-BY 4.0 International license.

Online supplemental figure 3

(A–L) Total cell number and turnover dynamics across all experimental conditions. (A) Flies maintained on a high-yeast (HY) diet throughout show only mild fluctuations in total cell number. These changes reflect moderate tissue turnover, with a progressive increase in the proportion of newly generated cells in the epithelium. (B) Infected flies kept on the HY diet before and after infection exhibit an initial decrease in total cell number, reaching a minimum at 12 hours post-infection. This is followed by marked regrowth, often surpassing pre-infection levels. (C) Flies maintained continuously on a high-sugar (HS) diet undergo gradual cell loss, with minimal production of new cells to compensate. (D) Infected flies kept on the HS diet before and after infection show more pronounced cell loss than uninfected controls, but this is partially offset by the production of new enterocytes, resulting in total cell numbers similar to unchallenged (UC) controls. (E) Flies initially maintained on HY diet and then shifted to HS diet display progressive cell loss. However, in contrast to flies constantly on HS (panel C), this loss is partially buffered by the production of new cells. (F) When HY-fed flies are shifted to HS diet following infection, total cell number drops sharply post-infection and then stabilizes. Despite this, tissue turnover remains elevated. (G) Flies initially on HS and then shifted to HY diet maintain stable total cell numbers and exhibit mild turnover, comparable to the HY-only condition in panel A. (H) Flies kept on HS prior to infection and shifted to HY post-infection display a mild drop in cell number, followed by substantial regrowth at 240 hours post-infection, along with elevated tissue turnover. (I) Flies initially on HY diet and then shifted to a starvation diet experience progressive cell loss with almost no turnover. All flies die by 48 hours post-treatment, the final recorded time point. (J) When HY-fed flies are shifted to starvation after infection, they exhibit a sharp initial drop in total cell number, accompanied by extensive tissue turnover. Again, no flies remain alive beyond 48 hours post-infection. (K) Flies initially kept on HS and then shifted to starvation maintain stable total cell numbers until succumbing to starvation. (L) Infected flies kept on HS and then moved to starvation show a slight reduction in total cell number and low levels of turnover before dying, with 48 hours post-infection being the last time point with surviving flies. All bar plots show RFP-positive (old) cells in red and RFP-negative (new) cells in blue, across time points. Statistical analyses compare treatments within the same condition for total cell number, RFP-positive cells (old), and RFP-negative cells (new).

bioRxiv preprint doi: https://doi.org/10.1101/2025.09.17.676798; this version posted September 17, 2025. The copyright holder for this preprint (which was not certified by peer review) is the author/funder, who has granted bioRxiv a license to display the preprint in perpetuity. It is made available under aCC-BY 4.0 International license.

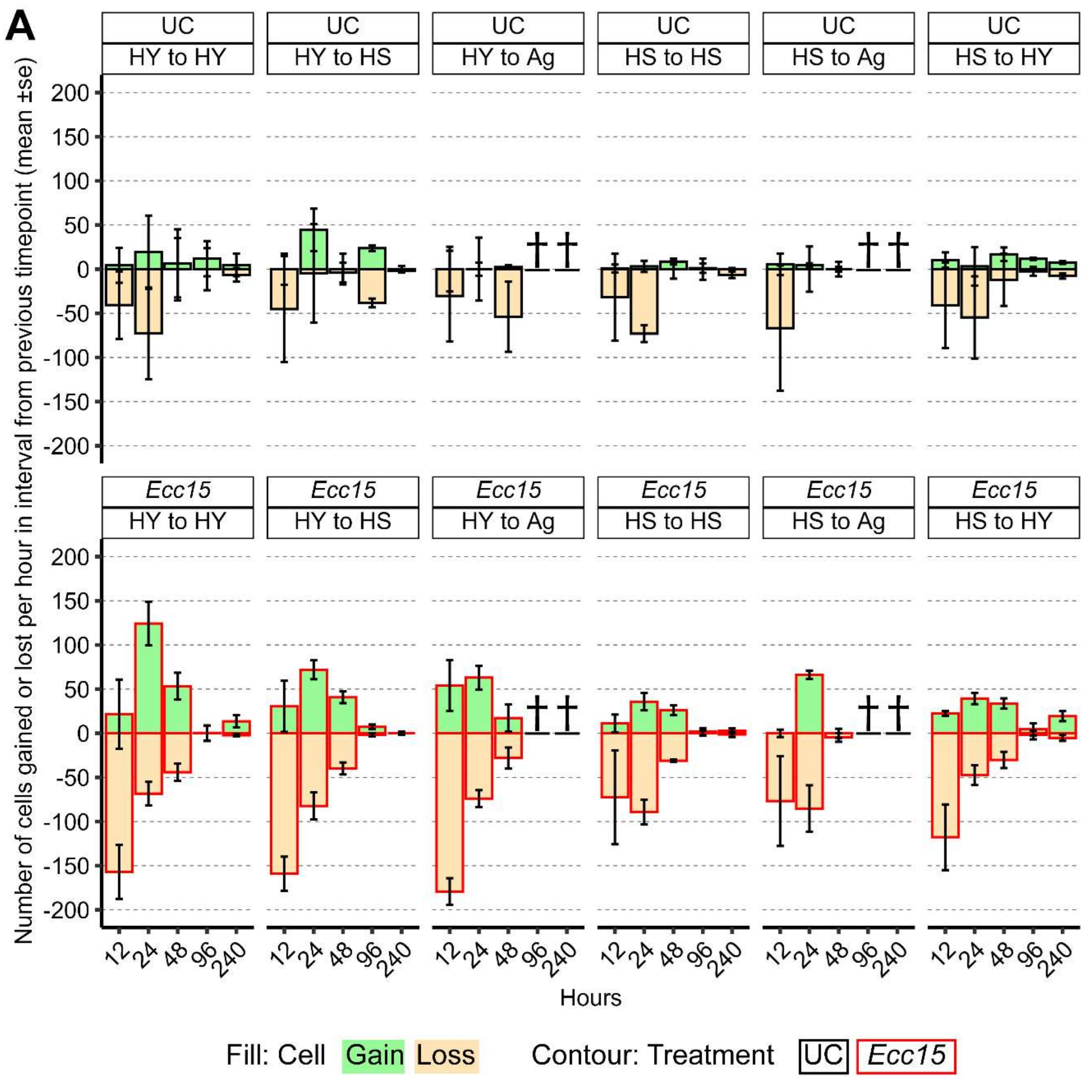

Online supplemental figure 4

A) Number of cells gained or lost per hour, in intervals from previous timepoint, show that while unchallenged samples have a relatively flat distribution (top part of the chart), infection with *Ecc15* greatly induced cell loss at 12 hours for most conditions, then decreased gradually (bottom part of the chart). Cell gain follows closely the kinetic of loss, with a slight delay. All facets of the bar plot show cells lost per hour in yellow and gained in green, across time points. Bar plot contour indicates treatment (UC = black, *Ecc15* = red).

bioRxiv preprint doi: https://doi.org/10.1101/2025.09.17.676798; this version posted September 17, 2025. The copyright holder for this preprint (which was not certified by peer review) is the author/funder, who has granted bioRxiv a license to display the preprint in perpetuity. It is made available under aCC-BY 4.0 International license.

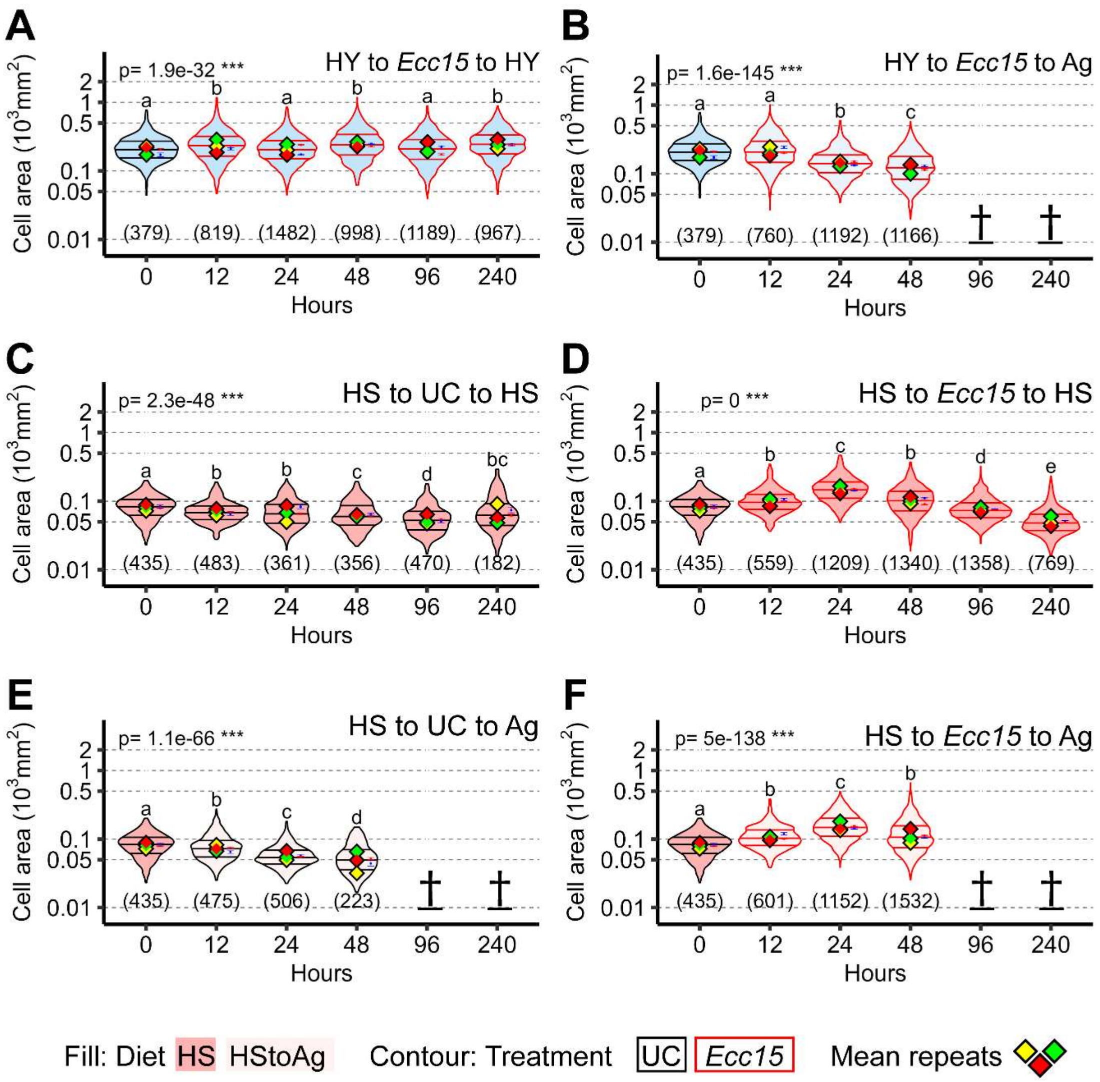

Online supplemental figure 5

A-B) Infection does not reduce cell size in midguts starting from HY in several separate experiments. C-F) In midguts starting from HS diet (small) and kept on either HS or Ag after treatment, infection also does not lead to a shrinkage in cell size, but leads to a transitory increase (D, F), before eventually shrinking down due to the effect of diet, as visible also in UC samples (C, E). For the violin/dot plots shown in this figure, colored lozenges represent means of replicate experiments. Black line connects the total mean of each sample to show timeline of changes. Violin plot fills are color-coded according to diets (HS = red, HY = light blue, HY to HS = pink, HS to HY = purple) and their contour indicates treatment (UC = black, *Ecc15* = red). Numbers in parentheses at the bottom of charts indicate sample sizes. p indicates the result of ANOVA for

bioRxiv preprint doi: https://doi.org/10.1101/2025.09.17.676798; this version posted September 17, 2025. The copyright holder for this preprint (which was not certified by peer review) is the author/funder, who has granted bioRxiv a license to display the preprint in perpetuity. It is made available under aCC-BY 4.0 International license.

samples in a single chart, and groups are obtained with Tukey Post Hoc test (letters above violins).

bioRxiv preprint doi: https://doi.org/10.1101/2025.09.17.676798; this version posted September 17, 2025. The copyright holder for this preprint (which was not certified by peer review) is the author/funder, who has granted bioRxiv a license to display the preprint in perpetuity. It is made available under aCC-BY 4.0 International license.

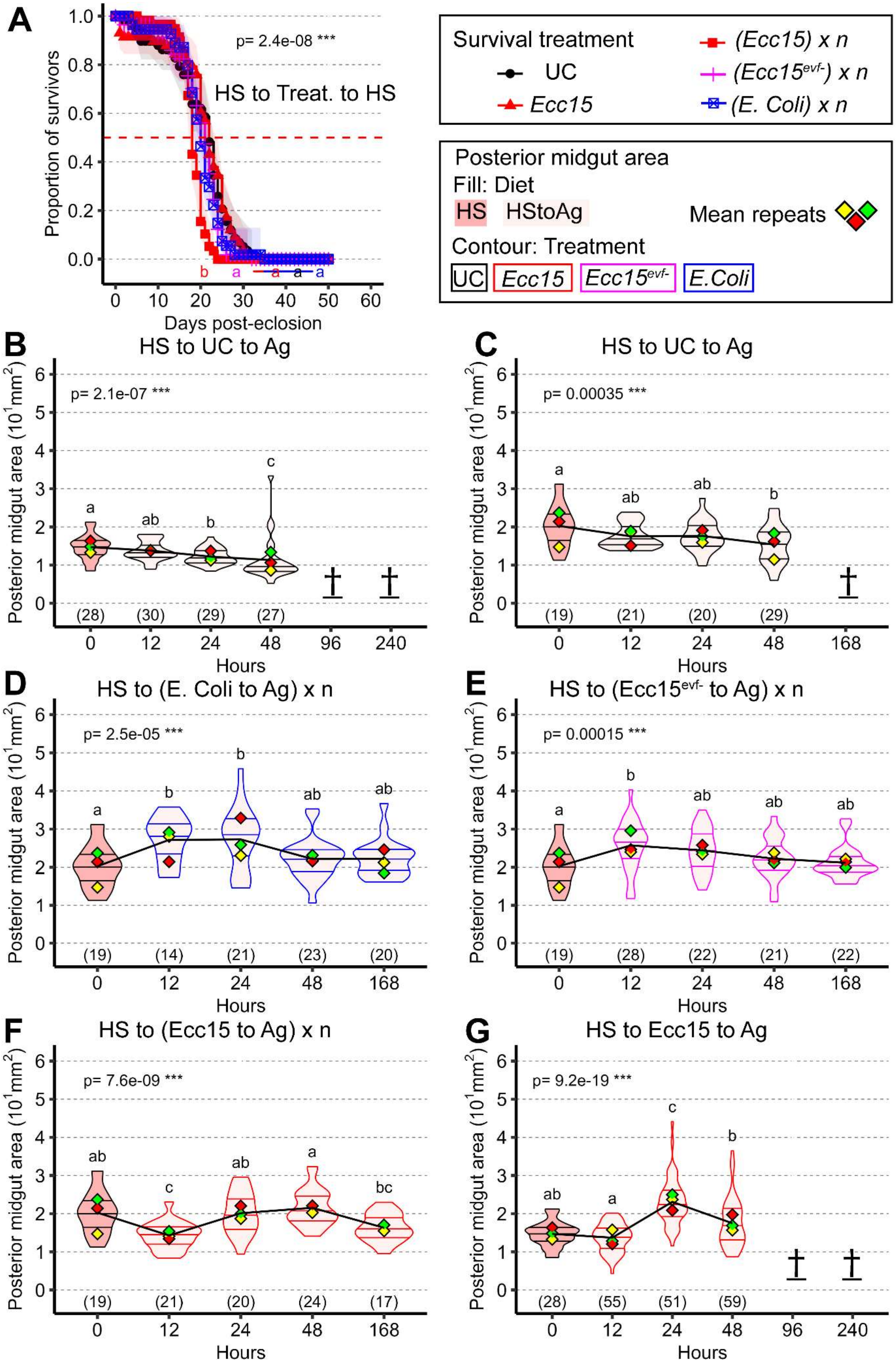

bioRxiv preprint doi: https://doi.org/10.1101/2025.09.17.676798; this version posted September 17, 2025. The copyright holder for this preprint (which was not certified by peer review) is the author/funder, who has granted bioRxiv a license to display the preprint in perpetuity. It is made available under aCC-BY 4.0 International license.

Online supplemental figure 6

A) Survival for flies always kept on HS with different cyclical treatments shows that repeated infection with *Ecc15* in this case leads to a small decrease in survival compared to the rest of the samples. B-G) Midguts of flies shifted from HS diet to Ag keep a relatively small size, with further shrinkage upon prolonged stay on Ag diet and eventually early demise at 48 hours (B original panel of experiment, C control for microbial addiction). Similarly to samples always on HS, repeated addition of nonpathogenic microbes (*E.Coli*, D and *Ecc15*$^{evf-}$, E) have an immediate effect in increasing the size of the midgut. Repeated infection with *Ecc15* in this condition does not lead to an increase in size of the midgut (F). If infected once with *Ecc15* before being shifted to Ag (G), flies show a transitory increase in midgut size at 24 hours post infection, followed by shrinkage and death in a similar manner as UC samples. This pattern repeated for a CantonS strain. For the violin/dot plots shown in this figure, colored lozenges represent means of replicate experiments. Black line connects total means of each sample to show timeline of changes. Violin plot fills are color-coded according to diets (HS = red, HY = light blue, HY to HS = pink, HS to HY = purple) and their contour indicates treatment (UC = black, *Ecc15* = red). Numbers in parentheses at the bottom of charts indicate sample sizes. p indicates the result of ANOVA for samples in a single chart, and groups are obtained with Tukey Post Hoc test (letters above violins). Survival statistics were calculated via Cox proportional hazards mixed effects model using the coxme package in R.
